# Increased Environment-Related Metabolism and Genetic Expression in the *In Vitro* Matured Mouse Oocytes by Transcriptome Analysis

**DOI:** 10.3389/fcell.2021.642010

**Published:** 2021-02-11

**Authors:** Hao-Lin Zhang, Yi Xu, Jia-Qian Ju, Zhen-Nan Pan, Jing-Cai Liu, Shao-Chen Sun

**Affiliations:** College of Animal Science and Technology, Nanjing Agricultural University, Nanjing, China

**Keywords:** meiosis, oocyte, *in vitro* maturation, gene expression, transcriptome analysis

## Abstract

Infertility in humans at their reproductive age is a world-wide problem. Oocyte *in vitro* maturation (IVM) is generally used in such cases to acquire the embryo in assisted reproductive technology (ART). However, the differences between an *in vivo* (IVO) and IVM culture environment in the RNA expression profile in oocytes, remains unclear. In this study, we compared the global RNA transcription pattern of oocytes from *in vitro* and *in vivo* maturation. Our results showed that 1,864 genes differentially expressed between the IVO and IVM oocytes. Among these, 1,638 genes were up-regulated, and 226 genes were down-regulated, and these changes were mainly divided into environmental adaption, metabolism, and genetic expression. Our detailed analysis showed that the expression of genes that belonged to metabolism-related processes such as energy metabolism, nucleotide metabolism, and carbohydrate metabolism was changed; and these genes also belonged to organismal systems including environmental adaptation and the circulatory system; moreover, we also found that the relative gene expression of genetic expression processes, such as protein synthesis, modification, and DNA replication and repair were also altered. In conclusion, our data suggests that *in vitro* maturation of mouse oocyte resulted in metabolism and genetic expression changes due to environmental changes compared with *in vivo* matured oocytes.

## Introduction

Oocyte meiotic maturation is an important process of oocyte quality control. This process is initiated during the germinal vesicle (GV) stage, and the oocyte undergoes germinal vesicle breakdown (GVBD) and metaphase I, and then is arrested at metaphase II until fertilization (Greenstein, 2005). Most basic research for oocyte maturation is based on the *in vitro* maturation (IVM) approach (Coticchio, 2016). For human reproduction, due to the high infertility rates worldwide, *in vitro* maturation is also widely applied in clinics of reproductive medicine centers (Cha and Chian, 1998). Since the first successful treatment with conventional *in vitro* maturation (IVM), assisted reproductive technology (ART) has become an integral part of modern medicine (De Geyter, 2019), and immature oocytes *in vitro* maturation (IVM) is currently one of the most advanced ART technologies (Zhao et al., 2020). IVM is an effective treatment that has yielded significant results with significant pregnancy and implantation rates (Son et al., 2019). To date, thousands of babies have been born using the IVM procedure, and a few clinical follow-up studies have shown no significant abnormalities in these babies compared with those conceived using conventional IVM treatment (Chian et al., 2014; Yang and Chian, 2017).

In spite of this, owing to culturing with a nutrient solution, the process of IVM has long been suspected of introducing unexpected modifications that may or may not adversely affect oocytes, which may subsequently manifest in embryonic development (Ye et al., 2020). The commercialized nutrient solution can not completely simulate the internal environment, which means that *in vitro* maturation or development may have a number of differences compared to IVO. It has been shown that the *in vitro* matured oocytes present distinct expression patterns which reflect the oocyte response to its surrounding environment (Katz-Jaffe et al., 2009). In porcine oocytes, *in vitro* maturation influences oocyte morphogenesis, which may also be recognized as significant mediators of cellular maturation capacity in pigs (Budna et al., 2017). A previous study illuminates the compensatory mechanism from a metabolic perspective during human oocyte maturation (Zhao et al., 2019). In addition, there are several differences in the modification of genes between culturing oocytes *in vitro* or *in vivo*. In a small-scale study of human IVM oocytes, one quarter is found to have an altered methylation pattern of the H19 differentially methylated region, which is normally unmethylated in *in vivo* matured oocytes (Borghol et al., 2006). Until now, whether culture conditions induce epigenetic alterations to deregulate gene expression remains contentious (Lu et al., 2018). The difference of the RNA transcriptome expression profile in mouse oocytes during maturation *in vivo* and *in vitro* remains unclear.

In cells, adaptive thermogenesis is a catabolic process that consumes energy-storing molecules and converts the energy into heat in response to environmental changes (Lynes et al., 2019). For example, Atp5e is involved in the biogenesis of ATP synthase, and knockdown of Atp5e produces an insufficient ATP phosphorylating capacity (Havlickova et al., 2010). Numerous supernumerary subunits of mitochondrial respiratory chain complex I (CI), Ndufb11, and Ndufa7, have been found to be targets of phosphorylation by cAMP-dependent protein kinase (mtPKA) and are thus likely to play a part in the regulation of CI activity and biogenesis (Rak and Rustin, 2014). These biological processes are all important for cells to adapt to the environment.

Thermogenesis is regulated by glucose metabolism, while it is has been shown that glucose metabolism is critical for determining oocyte developmental competence (Sutton-McDowall et al., 2010). The performance of the cell culture processes, in terms of both productivity and quality, is significantly influenced by cellular metabolism (Yuan et al., 2013; Rak and Rustin, 2014). Metabolic pathways contain multiple processes, such as glycolysis, fatty acid oxidation, glutamine metabolism (Rohlenova et al., 2018). In these processes, Ndufv3 as an accessory subunit of mitochondrial complex I participates in oxidative phosphorylation (Guerrero-Castillo et al., 2017). In addition, a previous study demonstrated that Cox7b was indispensable for cytochrome C oxidase (COX) assembly, COX activity, and mitochondrial respiration (Indrieri et al., 2012). Umps (uridine monophosphate synthase) was associated with pyrimidine metabolism, while the deficiency of Umps could cause orotic aciduria (OA) (Wortmann et al., 2017). Moreover, Ldhb (Lactate Dehydrogenase B) is involved in pyruvate metabolism by converting lactate to pyruvate (Urbanska and Orzechowski, 2019). These above-mentioned processes are vital components of metabolism.

Metabolism plays an important role in regulating gene expression, protein translation, and protein modification. The genetic expression of a cell, which includes DNA replication and repair, protein synthesis and modification, and other processes, is the kernel of cellular development. Several genes have been shown to be involved in genetic expression processing. 60S ribosomal protein L35 (RPL35) is an important part of the 60S ribosomal subunit, which has a role in protein translation and endoplasmic reticulum (ER) docking (Jiang et al., 2015). A study also found that the loss of zebrafish ns or gnl2 has a major impact on 60S large ribosomal subunit formation and/or function due to cleavage impairments at distinct sites of the pre-rRNA transcript (Essers et al., 2014). The Polymerase delta (POLD1) gene participates in providing the essential catalytic activities of polymerase δ (Polδ), which is essential for replication (Nicolas et al., 2016).

Here we investigated the transcriptome characteristics of mouse oocytes matured *in vitro* to gain a transcriptome-level understanding of the differences between mouse IVM and IVO matured oocytes. We used RNA-seq to screen differentially expressed genes through KEGG, GO, and other analytical methods. We showed that *in vitro* matured oocytes had a different mRNA expression profile from an environmental change response, cellular metabolism, and genetic expression aspect.

## Materials and Methods

### Oocyte Collection and Culture

All procedures with mice were conducted according to the guidelines issued by the Animal Research Institute Committee of Nanjing Agriculture University, China. All the mice used in the experiment were purchased from Qinglongshan Animal Farm in Nanjing. This committee approved the experimental protocols. *In vitro* oocytes were collected from 6- to 8-week-old ICR mice and cultured in M2 medium under paraffin oil at 37°C in an atmosphere containing 5% CO^2^. *In vivo* oocytes were collected from the tubal ampulla of 6- to 8-week-old ICR mice. And then each group of oocytes were preserved in lysis buffers provided by Geekgeen in Beijing. Each tube of lysis buffer consists of 350 μL A and 3.5 μL B, abiding by the principle of diluting it when it is used.

### RNA-seq Analysis (Transcriptome Sequencing)

Transcriptome sequencing was performed on the IVO group and the IVM group mouse oocytes. We collected 80 oocytes for each group for disposing using Beijing Geek Gene Technology Co. Ltd. The total RNA was first extracted using the RNeasy Micro Kit (QIAGEN, 74004, Quigen, Toronto, Canada) and then quantified by the Qubit RNA Assay Kit T (Invitrogen, Eugene, OR, USA). The protocol was constituted by sequential RNA fragmentation, reverse transcription using random primers, second strand cDNA synthesis, end repair, dA-tailing, adapter ligation, and PCR enrichment. The concentration and quality of libraries were measured by a NanoDrop 2000 spectrophotometer (Thermo Scientific, Wilmington, DE, USA), Q-PCR, and Agilent 2100 Bioanalyzer (Agilent Technologies, Palo Alto, CA, USA). A 150bp anti-terminal sequence was then performed on the library. Filtering out low quality sequences and joint sequences, the Clean Reads were analyzed. Hierarchical clustering was carried out at the gene level, and the data of all sample genes were calculated according to the log_2_(FPKM+1). The desired genes were selected and compared to determine the changes in the expression levels of the relevant genes. RNA library sequencing was performed on the Illumina HiseqTM 2500/4000 by Gene Denovo Biotechnology Co., Ltd (Guangzhou, China).

### RNA Isolation and Quantitative Real-Time PCR

Mouse oocytes maturated *in vitro* cultured for 12 h, and *in vivo* matured oocytes were obtained from ampullar potion. The oocytes in the IVO group and the IVM group were collected. Total RNA was extracted from exactly 30 oocytes with a Dynabead mRNA DIRECT kit (Invitrogen Dynal, Oslo, Norway). According to the manufacturer's instructions (Invitrogen), the first strand was synthesized with a cDNA synthesis kit (Takara) by Oligo (dT) 12–18 primers. The cDNA was stored at −20°C until analysis. The levels of relevant mRNAs were determined by quantitative RT-PCR using a FastStart Universal SYBR Green Master (Rox; Roche Applied Science, Mannheim, Germany) with One plus Real-Time PCR System (Applied Biosystems, Life Technologies, Carlsbad, CA, USA). Gene expression levels were analyzed using the 2–ΔΔCt method after the melting-curve analysis was completed. The expression levels of the target genes were then normalized to the expression level of GAPDH in each sample. The primers are listed in Supplementary Table 1.

### Statistical Analysis

For each treatment, at least three valid replicates were tested, and the results are reported as mean ± SEM. Statistical comparisons were made by independent-sample *t*-tests using GraphPad Prism 5 statistical software. Results were considered significant at *P* < 0.05. Bioinformatic analysis was performed using Omicsmart, a real-time interactive online platform for data analysis (http://www.omicsmart.com).

## Results

### Global Transcriptomes Characteristics Between Mouse IVM and IVO Oocytes

**Figure 1:**
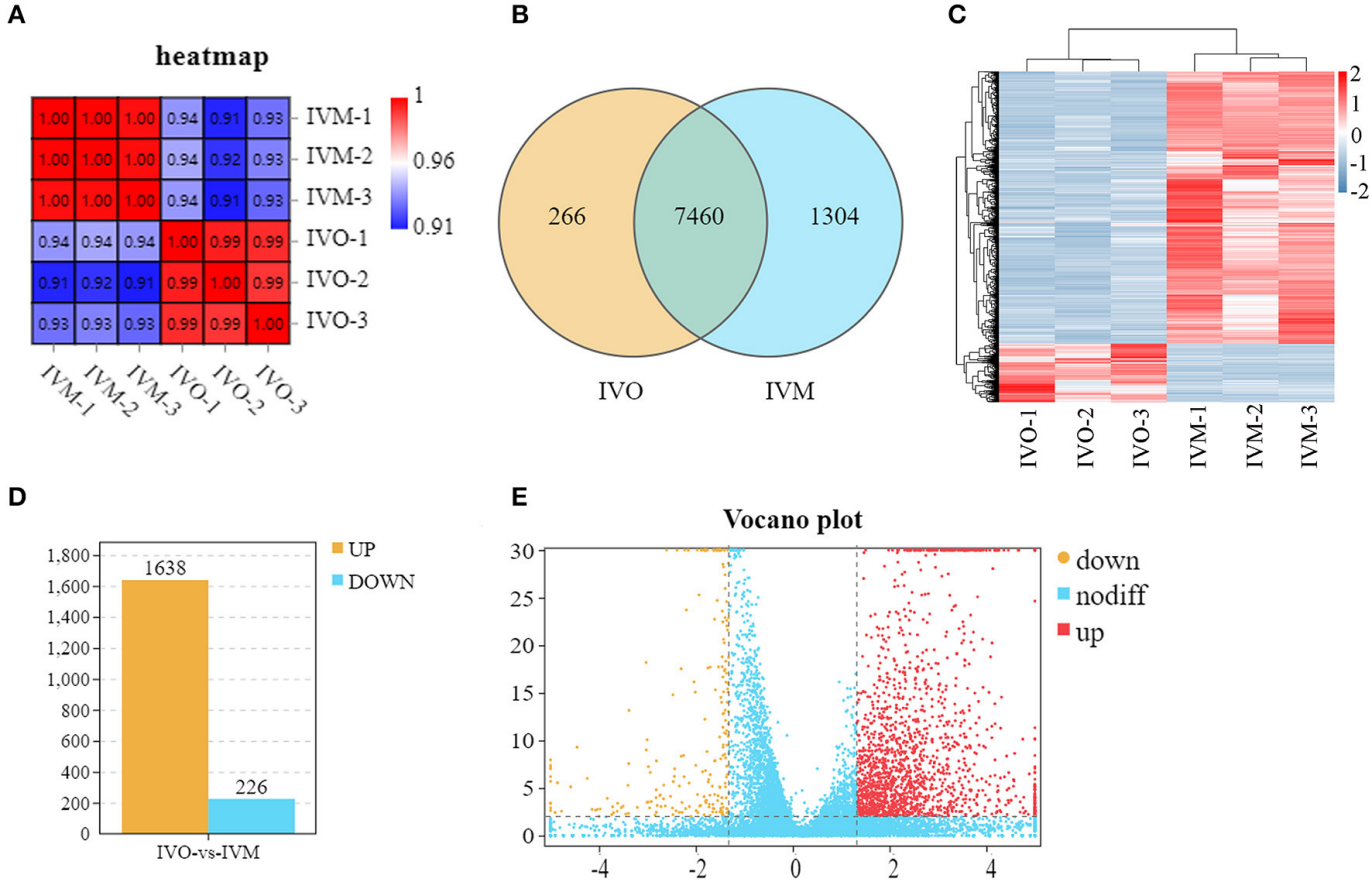
Global transcriptomes characteristics between mouse IVM and IVO oocytes. **(A)** Correlation heatmap showing the repeatability between repetitive samples and the difference between groups. **(B)** Venn diagram showing the number of DEGs in common among the two samples. **(C)** Mouse IVM and IVO oocytes can be clearly distinguished based on their transcriptome characteristics. The color key (from blue to red) of Z-score value (−2–2) indicated low to high expression levels. **(D)** The statistical analysis of differentially expressed genes between IVO and IVM oocytes. **(E)** The volcano analysis of differentially expressed genes with color-coded. The gene with *Q* < 0.01 and FC > 2.5 is marked in red; the gene with *Q* < 0.01 and FC < −2.5 is marked in yellow; the gene with *Q* > 0.01 and −2.5 < FC <2.5 is marked in blue.

We used a transcriptome analysis to identify the different expression levels of mRNA between IVO and IVM oocytes and the Pearson correlation coefficient between each of the two samples was calculated. As shown in Figure 1A, the heatmap indicated that the intraclass samples had a high level of repeatability. We then counted the overlapping genes in the IVO group and IVM group, and we found that there are 7,460 genes detected in both groups (Figure 1B). Samples from the IVM and IVO groups were further distinguished by supervised hierarchical clustering (Figure 1C). Subsequently, 1,864 differentially expressed genes (DEGs) were screened out by log_2_(Fold Change) ≥2.0, among which 1,638 genes were highly expressed in the IVM group and 226 genes were highly expressed in the IVO group (Figure 1D) This is also shown by the data analysis of the volcano plot (Figure 1E). Therefore, we show that IVM oocytes have significantly differentially expressed genes compared with IVO oocytes.

### GO and KEGG Analysis of DEGs for Mouse IVM and IVO Oocytes

**Figure 2:**
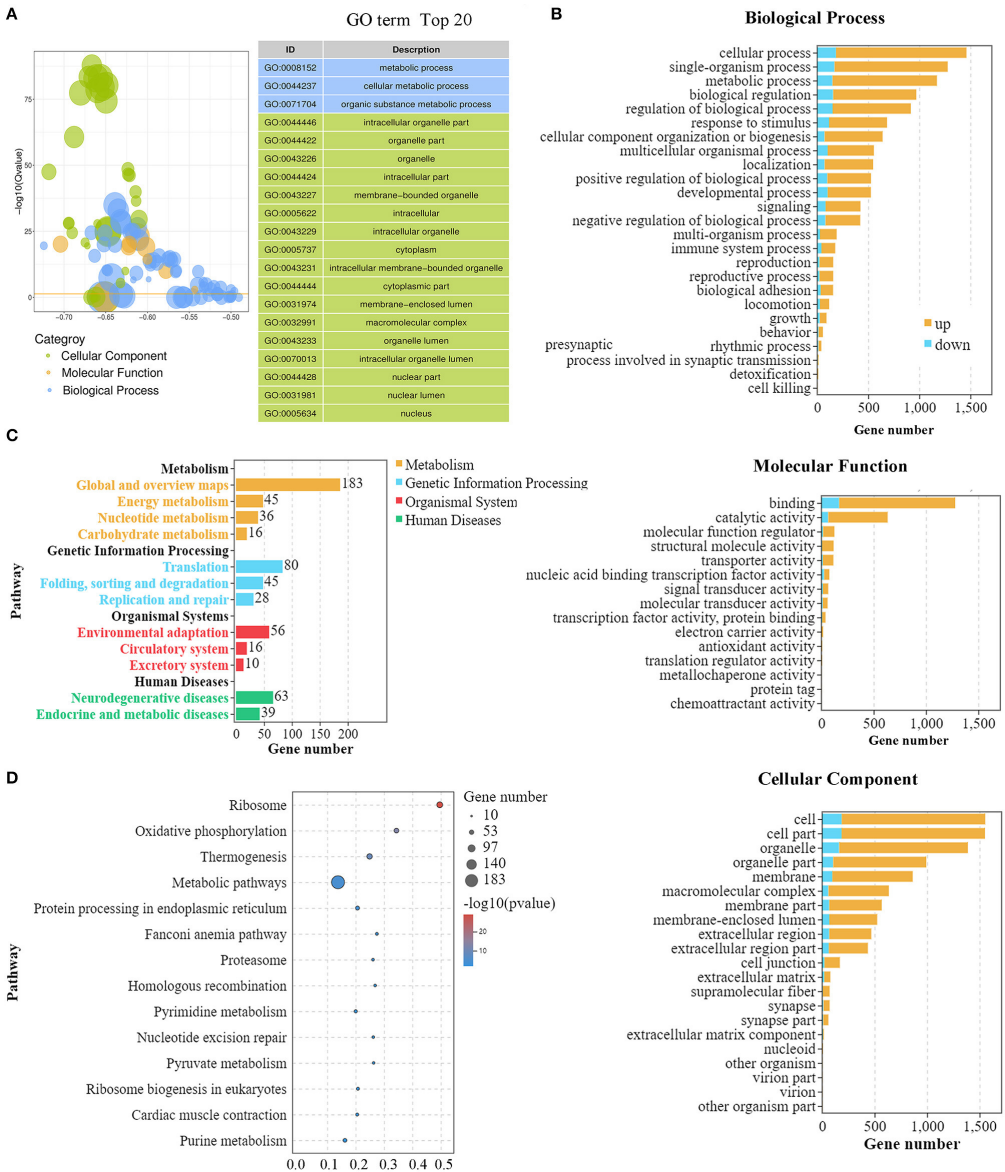
GO and KEGG analysis of DEGs for mouse IVM and IVO oocytes. **(A)** GO summary graph showing the summary of the top 20 enriched GO items. Different colors represent different GO categories. **(B)** The main biological process, cellular component, and molecular function of DEGs between mouse IVM and IVO oocytes. It shows the number of up- and down-regulated genes gathering in different classifications. **(C)** The KEGG analysis of DEGs based on classification by pathways. Each column represents a pathway, and the height of the column represents the number of genes that pathway contains. Legends indicate the pathway represented by different column colors. **(D)** The rich factors by the KEGG enrichment analysis for the differentially expressed genes of IVM treatment.

We then used Gene Ontology (GO) and KEGG analysis to further sift the significantly DEGs between the IVO and IVM groups. As shown in Figure 2A, the top 20 GO terms, classified by –log_10_(Q value), were significantly enriched in DEGs compared to the genome background Gene Ontology which is an international standardized gene functional classification system. It has three ontologies including the biological process (BP), molecular function (MF), and the cellular component (CC). In terms of the biological process, cellular process, and metabolic process, the biological regulation and regulation of the biological process were shown to be interfered; for the molecular function, binding and catalytic activity showed a significantly close relationship; for the cellular component, cell, cell part, organelle and organelle part were revealed with salient differences (Figure 2B). Furthermore, the KEGG enrichment analysis displayed the classification of the top 20 pathways which revealed that these differentially expressed genes were mainly concentrated in four KEGG A classes including metabolism, genetic information processing, organismal systems, and human diseases, which are sorted by pathway type (Figure 2C). Among these pathways, five pathways including oxidative phosphorylation, pyrimidine metabolism, pyruvate metabolism, purine metabolism, and the citrate cycle were related to metabolism; seven pathways including ribosome, protein processing in ER, Fanconi anemia pathway, proteasome, homologous recombination, nucleotide excision repair, and ribosome biogenesis in eukaryotes were involved in genetic expression; three pathways including thermogenesis, cardiac muscle contraction, and vasopressin-related water reabsorption were related to organismal systems; and four pathways covering Parkinson disease, Huntington disease, Alzheimer disease and Non-alcoholic fatty liver disease affected human diseases. A detailed graph shows that these genes were related with the first 14 pathways (Figure 2D).

### Increased Metabolic Pathways in IVM Oocytes Compared With IVO Oocytes

**Figure 3:**
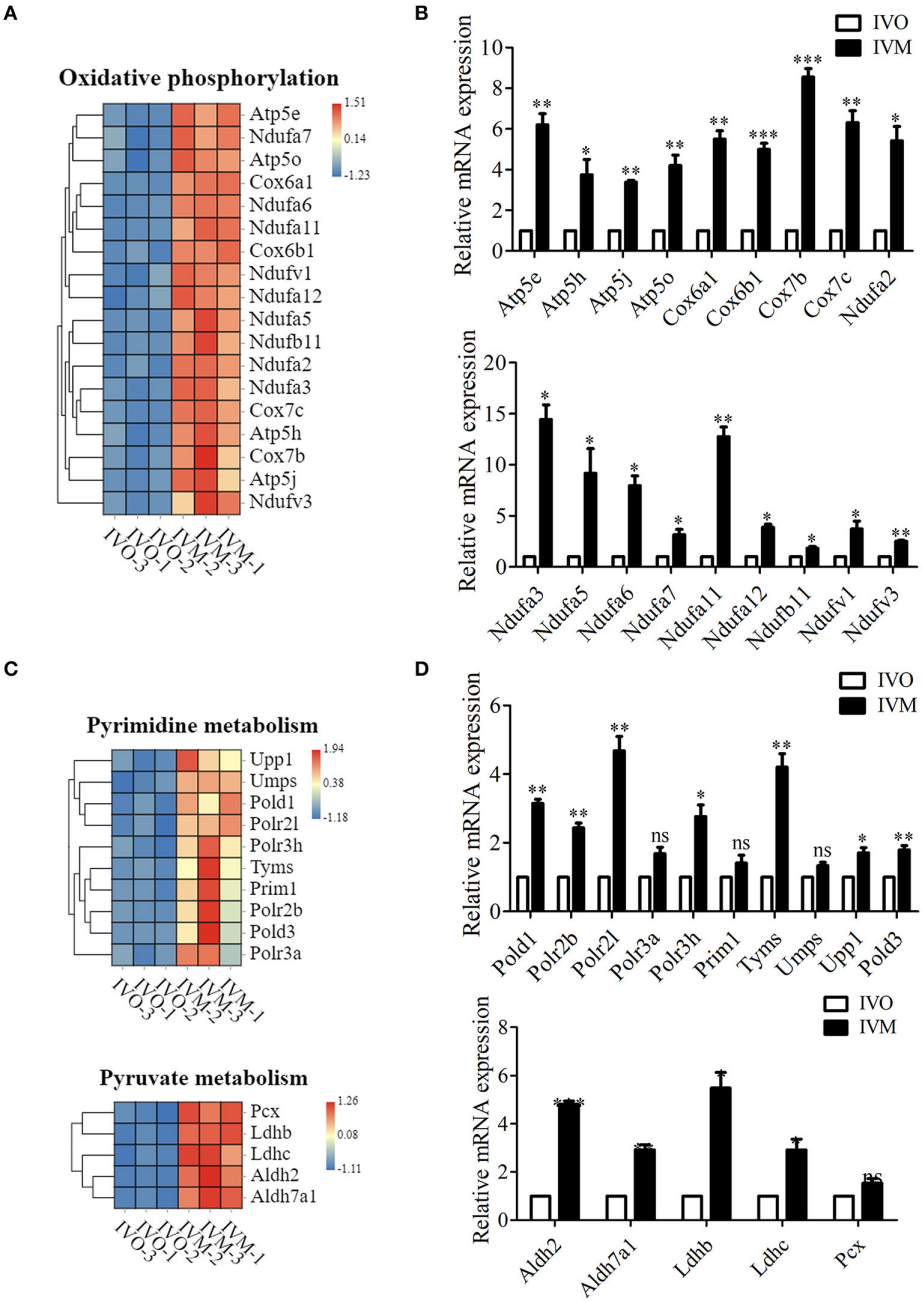
Increased metabolic pathways in IVM oocytes compared with IVO oocytes. **(A)** The heat map for the gene expression level of oxidative phosphorylation pathway. **(B)** Relative mRNA expression levels of genes involved in oxidative phosphorylation in both IVM and IVO oocytes. *p < 0.05; **p < 0.01; ***p < 0.001. **(C)** The heat map for the gene expression level of pyrimidine metabolism and pyruvate metabolism pathways. **(D)** Relative mRNA expression levels of genes regulated the pyrimidine metabolism and pyruvate metabolism pathways in IVM and IVO oocytes. *p < 0.05; **p < 0.01; ***p < 0.001.

It has been shown that glucose concentration in the *in vitro* culture environment could affect oocyte metabolic pathways (Sutton-McDowall et al., 2010). In the KEGG column diagram, we also found that the pathways related to metabolism, like energy metabolism, nucleotide metabolism and carbohydrate metabolism fluctuated. We first analyzed the oxidative phosphorylation-related genes, and the results showed that more than 45 genes were up-regulated in the IVM oocytes compared with IVO oocytes (Figure 3A). To further verify this, we examined these gene expressions through quantitative real-time PCR, and the results showed that the genes, Ndufv3, Ndufa5, Ndufa12, etc., were all increased compared with IVO oocytes (Figure 3B). Moreover, we also found that the pyrimidine-related genes, such as Upp1, Umps, Polr2l, which were related with nucleotide metabolism, also showed different expressions compared with IVO oocytes (Figure 3C), and this was also confirmed by the quantitative real-time PCR results (Figure 3D). Likewise, the expression of more than 16 carbohydrate metabolism-related genes were altered, and several prominent genes, including Pcx, Ldhb, Ldhc, Aldh2, and Aldh7a1 showed increased expression (Figure 3C). We also performed the mRNA expression validation, and the result was consistent with the analysis above (Figure 3D). Therefore, our results showed that *in vitro* maturation could give rise to increased expression of metabolic pathways.

From the KEGG analysis results above we found that several pathways related to environmental adaptation, the circulatory and excretory system were altered. While adaptive thermogenesis converts the energy into heat in response to environmental changes (Lynes et al., 2019), we then further analyzed the thermogenesis-related genes, and the results showed that more than 19 genes in the IVM oocytes showed a difference compared with IVO oocytes (Supplementary Figure 1A). To further confirm this, we examined the mRNA expression level of these genes, and the results showed that the genes, Atp5e, Cox6a1, Cox7b, etc., were all increased compared with IVO oocytes (Supplementary Figure 1B). Moreover, we also found that the cardiac muscle contraction-related genes, such as Cox6a1, Cox6b1, Cacnb3, Cox7b, Cox7c, which are related with the circulatory system also showed differences (Supplementary Figure 1C), and this was also confirmed by the quantitative real-time PCR results (Supplementary Figure 1D). The altered genes indicated that the oocyte capacity related to environmental adaption was activated during *in vitro* maturation compared with *in vivo* maturation.

### Increased Genetic Information Processing in IVM Oocytes Compared With IVO Oocytes

**Figure 4:**
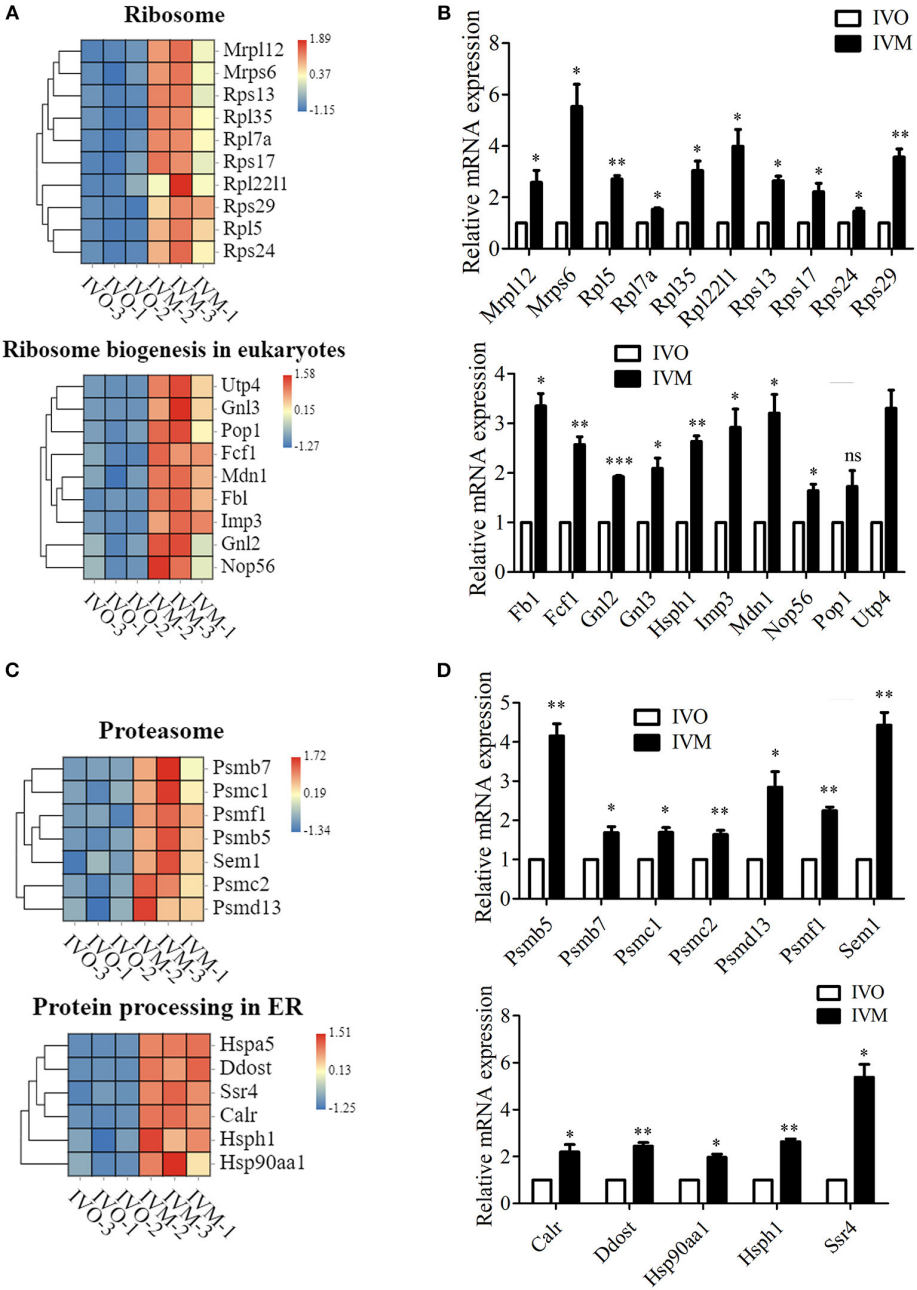
Increased genetic information processing in IVM oocytes compared with IVO oocytes. **(A)** The heat map for the gene expression level of ribosome and ribosome biogenesis in eukaryotes pathway. **(B)** Relative mRNA expression levels of genes that involved in ribosome and ribosome biogenesis in eukaryotes in both IVM and IVO oocytes. *p < 0.05; **p < 0.01; ***p < 0.001. **(C)** The heat map for the gene expression level of proteasome and protein processing in ER. **(D)** The validation of mRNA expression level for proteasome and protein processing in ER. *p < 0.05; **p < 0.01; ***p < 0.001.

**Figure 5:**
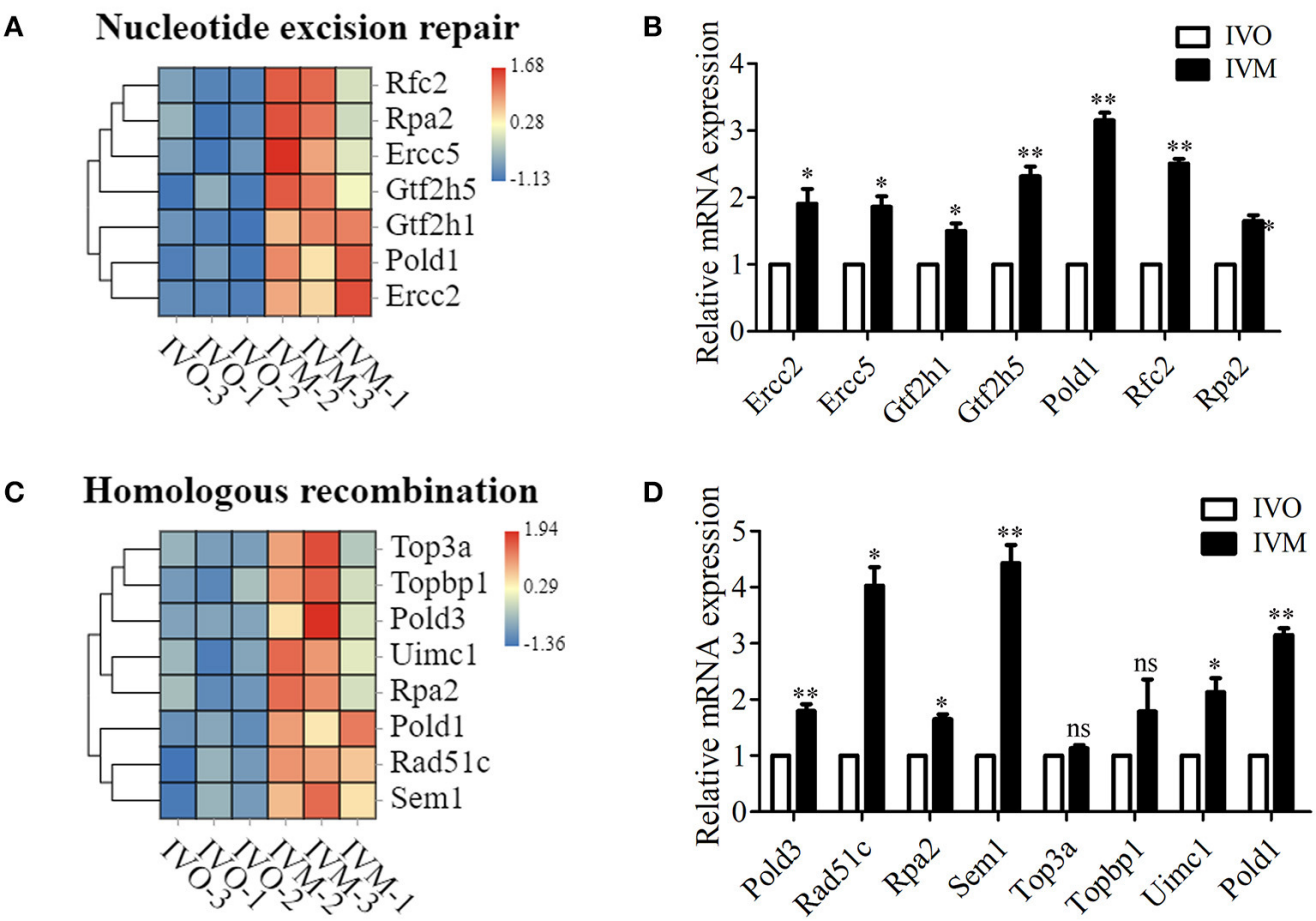
Increased genetic information processing in IVM oocytes compared with IVO oocytes. **(A)** The heat map for the gene expression level of DNA repaired-related genes. **(B)** Relative mRNA expression levels of genes that involved in nucleotide excision repair in both IVM and IVO oocytes. *p < 0.05; **p < 0.01. **(C)** The heat map for the gene expression level of homologous recombination. **(D)** Relative mRNA expression levels of genes that participated in homologous recombination in both IVM and IVO oocytes. *p < 0.05; **p < 0.01.

We next investigated the genetic expression-related genes. The KEGG pathway diagram showed that DNA replication and repair, protein synthesis and modification were affected. The analysis of ribosome and its biogenesis showed that more than 19 genes were up-regulated (Figure 4A), and the mRNA level of these genes were confirmed by the relative mRNA expression analysis (Figure 4B). Moreover, we also analyzed the protein degradation and modification processes, and the results showed that the expression of more than 13 genes were altered (Figure 4C). Real time RT-PCR data showed that the level of Psmb5, Psmb7, Psmc1, etc. expression increased in the IVM oocytes (Figure 4D). Afterwards, we performed statistical analysis of the DNA replication and repair-related genes, and the heat map showed that Rfc2, Ercc5, Top3a, Rpa2, etc. expressed differentially between these two groups (Figure 5A). We then carried out real time RT-PCR to confirm these results, and the mRNA expression level of Ercc2, Gtf2h5, and Pold1, which were related with DNA repair, showed the increase, which was consistent with the evaluation of the analysis results above (Figure 5B). Similarly, the mRNA expression level of Pold3, Rpa2, and Top3a, which were related with DNA replication, showed up-regulated characteristic, which conformed to the assessment of the analysis results above (Figures 5C,D). Therefore, we speculated that *in vitro* maturation affected the processing of genetic expression in mouse oocytes.

## Discussion

In this study we aimed to investigate the effects of *in vitro* maturation on mouse oocytes at the transcription level. We carried out our study by means of a transcriptome analysis to screen the differentially expressed genes. The genes were then grouped together to identify the biological processes that were affected. Our data showed that *in vitro* matured oocytes had multiple difference for the RNA expression compared with the *in vivo* maturation.

We first analyzed the dynamic changes in the global gene expression in mouse IVM and IVO oocytes. Our results showed that among the common differentially expressed genes of IVO and IVM groups, nearly 22% of the genes showed up-regulation expression in the IVM group, while only 3% was down-regulated. To further understand the influence of *in vitro* maturation on oocytes, we used the GO and KEGG analysis, starting with BP, MF, CC, and pathways respectively, to explore the issue. The analysis results showed that most genes related to environmental adaption were up-regulated, while metabolic processes were most affected, and the processes of genetic information, such as translation, folding, sorting and degradation, and replication and repair, were also altered. Compared with *in vivo* matured oocytes, the most striking difference of *in vitro* maturation processing was culturing conditions. As a result, we first detected the responses of oocytes to adapt environmental changes. Our results showed that thermogenesis, which was associated with the generation of energy, was answered more obviously. Previous studies showed that the environmental factors, such as light and culture medium composition, can induce metabolic alterations in the cytoplasm, resulting in mitochondrial dysfunction, augmentation of lipid peroxidation, and embryo growth arrest in cultured oocytes (Agarwal et al., 2006). While in human oocytes it was shown that *in vitro* maturation seemed to alter the mitochondrial membrane potential and endoplasmic reticulum (ER) compared with *in vivo* maturation (Ferrer-Vaquer et al., 2019). In addition, the mitochondrial activity was reduced in mature oocytes following 24 h of arrest and IVM (Santiquet et al., 2017). Part of the ultrastructure features in human oocytes shows abnormality *in vitro*, such as large mitochondria-vesicles (MV) complexes which partially replaced mitochondria-smooth endoplasmic reticulum (M-SER) aggregates in IVM oocytes (Coticchio et al., 2016). It has been demonstrated that *in vitro* culture conditions exert oxidative stress or an imbalance between oxidants and antioxidants, while the oxidative stress is generally resulted from mitochondrial dysfunction (Combelles et al., 2009). Our data provided the molecular explanation for these previous studies and indicated that *in vitro* maturation might disturb oxidative phosphorylation processing in mouse oocytes. The pyrimidine pathway is a vital metabolic pathway which yields in the formation of pyrimidines, that are then integrated in nucleic acids (DNA and RNA) in sugars (UDP sugars) and lipids (CDP lipids) (Tiwari and Dubey, 2018). Our results indicated that several genes of the pyrimidine metabolism pathway were affected. Similar results were found for the genes of pyruvate metabolism. *In vitro* meiosis induction by gonadotropin is dependent upon the presence of glucose (Fagbohun and Downs, 1992), and the beneficial effect of glucose on oocyte maturation takes place through its glycolysis to pyruvate (Downs et al., 1996). Silencing mitochondrial pyruvate carrier 1 of cumulus-denuded oocytes (DOs) significantly impaired their maturation (Xie et al., 2018). Therefore, our data reveals that *in vitro* maturation may cause damage to metabolism pathways, resulting in increased gene expression.

We also found that the genetic information processing had significantly different expression levels between IVM and IVO oocytes. Genetic information processing in relation to transcription and translation of genes, regulates the synthesis of proteins for oocytes and indirectly control vital activities. The *in vitro* maturation procedure strongly affects the gene expression profile of human oocytes, including several genes involved in transcriptional regulation, embryogenesis, epigenetics, development, and the cell cycle (Virant-Klun et al., 2018). *In vitro*-produced fresh (IVF-F) bovine blastocysts, gene expression of ribosome, and its biogenesis was significantly up-regulated (Aksu et al., 2012). In cattle, *in vitro* production (IVP) embryos may display aberrations in ribosomal RNA gene activation (Hyttel et al., 2000). Our analysis results were consistent with previous studies, confirming that *in vitro* maturation could impair protein synthesis, and caused an increase in gene expression as compensation. Additionally, studies indicated that ER stress had been identified in mammalian oocytes and embryos produced *in vitro*, while functional proteins must be folded properly in the endoplasmic reticulum (ER) to maintain oocyte and embryo development (Lin et al., 2019). In human oocytes, *in vitro* maturation may cause dysregulation in either gene transcription or post-transcriptional modification of genes with the over-abundance of transcripts (Jones et al., 2008).

In conclusion, our data, through transcriptome analysis, indicated that *in vitro* maturation might induce errors through multiple pathways including the generation of energy and gene expression processing in mouse oocytes.

## Data Availability Statement

The original contributions presented in the study are included in the article/Supplementary Material, further inquiries can be directed to the corresponding author/s. The original data GEO Submission (GSE165546) (NCBI tracking system #21628819) could be found at the following link: https://www.ncbi.nlm.nih.gov/geo/query/acc.cgi?acc=GSE165546.

## Ethics Statement

The animal study was reviewed and approved by Nanjing Agricultural University.

## Author Contributions

H-LZ and S-CS designed the study, wrote the manuscript, and analyzed the data. H-LZ performed the experiments. YX, Z-NP, and J-QJ contributed the materials. All the authors approved the final manuscript.

## Conflict of Interest

The authors declare that the research was conducted in the absence of any commercial or financial relationships that could be construed as a potential conflict of interest.

## Supplementary Material

Supplementary Figure 1 Increased environmental adaptation in the IVM oocytes compared with IVO oocytes. **(A)** The heat map for the gene expression level of thermogenesis pathway. **(B)** The verification for the mRNA expression of differentially expressed genes from thermogenesis pathway. ^*^*p* < 0.05; ^**^*p* < 0.01; ^***^*p* < 0.001. **(C)** The heat map for the gene expression level of cardiac muscle contraction pathway. **(D)** The verification for the mRNA expression of differentially expressed genes from cardiac muscle contraction pathway. ^*^*p* < 0.05; ^**^*p* < 0.01; ^***^*p* < 0.001.

## Abbreviations

IVM: *In vitro* maturation
IVO: *In vivo* maturation
ART: Assisted reproductive technology
GV: Germinal vesicle
GVBD: Germinal vesicle breakdown
MI: Metaphase I
MII: Metaphase II
DEGs: differentially expressed genes
GO: Gene Ontology
MV: Mitochondria-vesicles
M-SER: Mitochondria-smooth endoplasmic reticulum
DOs: cumulus-denuded oocytes.
