# Supplementary figures and images for: Increased Environment-Related Metabolism and Genetic Expression in the In Vitro Matured Mouse Oocytes by Transcriptome Analysis
Source: Front Cell Dev Biol. 2021 Feb 11;9:642010. doi: 10.3389/fcell.2021.642010 (PMC7928285; doi:10.3389/fcell.2021.642010)

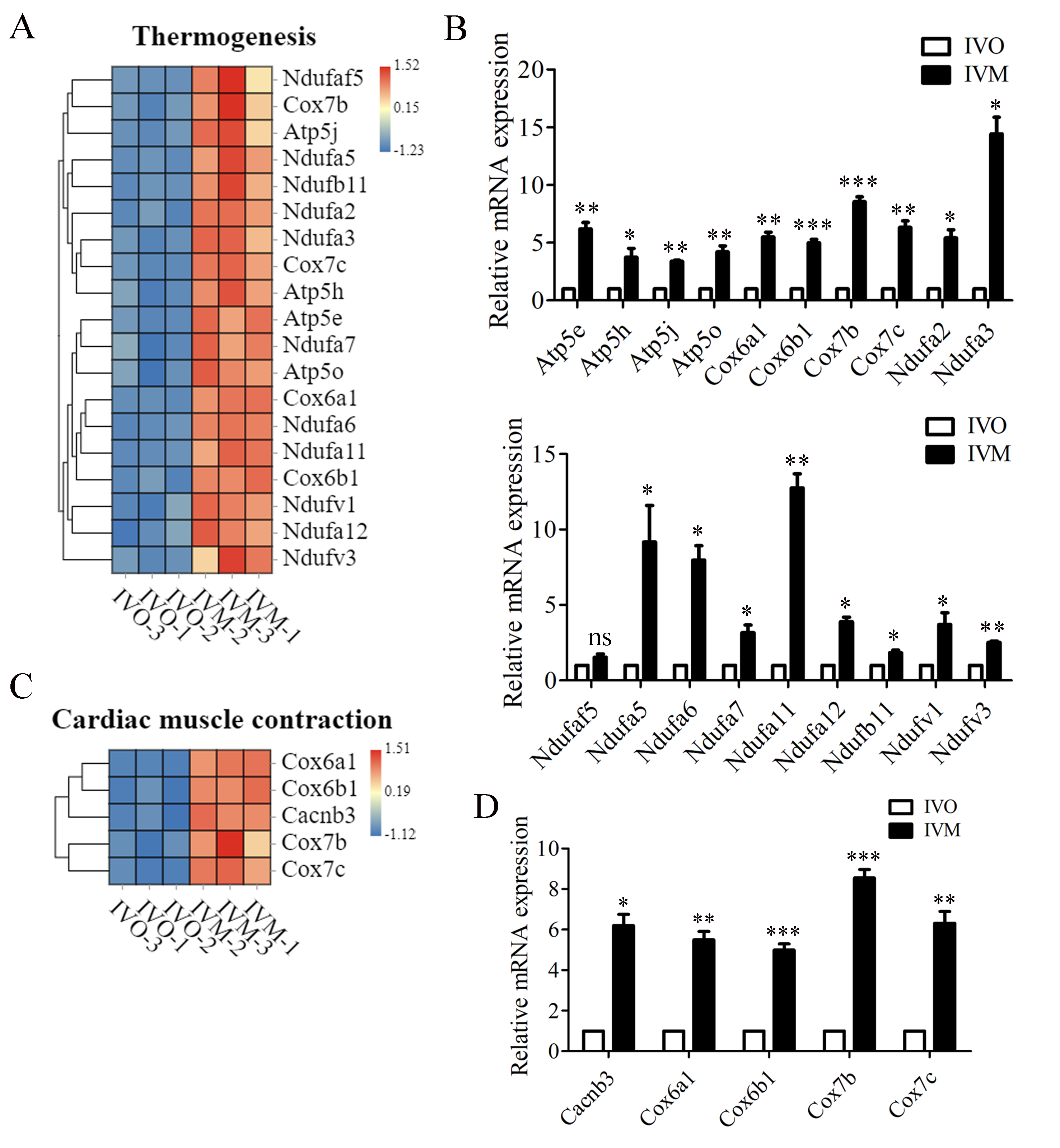

Supplement: Supplementary Figure 1 — Increased environmental adaptation in the IVM oocytes compared with IVO oocytes. (A) The heat map for the gene expression level of thermogenesis pathway. (B) The verification for the mRNA expression of differentially expressed genes from thermogenesis pathway. *p < 0.05; **p < 0.01; ***p < 0.001. (C) The heat map for the gene expression level of cardiac muscle contraction pathway. (D) The verification for the mRNA expression of differentially expressed genes from cardiac muscle contraction pathway. *p < 0.05; **p < 0.01; ***p < 0.001. [file Image_1.TIF]
